# Characterization of Rhinitis According to the Asthma Status in Adults Using an Unsupervised Approach in the EGEA Study

**DOI:** 10.1371/journal.pone.0136191

**Published:** 2015-08-26

**Authors:** Emilie Burte, Jean Bousquet, Raphaëlle Varraso, Frédéric Gormand, Jocelyne Just, Régis Matran, Isabelle Pin, Valérie Siroux, Bénédicte Jacquemin, Rachel Nadif

**Affiliations:** 1 INSERM, U1168, VIMA: Aging and chronic diseases, Epidemiological and Public health approaches, F-94807, Villejuif, France; 2 Univ Versailles St-Quentin-en-Yvelines, UMR-S 1168, F-78180, Montigny le Bretonneux, France; 3 University hospital, Montpellier, France; 4 CHU de Lyon, Pneumology Department, Lyon, France; 5 Allergology Department, Centre de l’Asthme et des Allergies, Hôpital Armand-Trousseau (APHP), APHP, Paris, France; 6 Université Paris 6 Pierre et Marie Curie, Paris, France; 7 Univ Lille Nord de France, F-59000, Lille, France; 8 INSERM, IAB, Team of Environmental Epidemiology applied to Reproduction and Respiratory Health, F-38000 Grenoble, France; 9 Univ. Grenoble Alpes, F-38000 Grenoble, France; 10 CHU de Grenoble, F-38000 Grenoble, France; 11 CHU de Grenoble, Pediatric Department, F-38000, Grenoble, France; 12 CREAL-Centre for Research in Environmental Epidemiology Parc de Recerca Biomèdica de Barcelona, Barcelona, Spain; Tongji Medical College, CHINA

## Abstract

**Background:**

The classification of rhinitis in adults is missing in epidemiological studies.

**Objective:**

To identify phenotypes of adult rhinitis using an unsupervised approach (*data-driven*) compared with a classical *hypothesis-driven* approach.

**Methods:**

983 adults of the French Epidemiological Study on the Genetics and Environment of Asthma (EGEA) were studied. Self-reported symptoms related to rhinitis such as nasal symptoms, hay fever, sinusitis, conjunctivitis, and sensitivities to different triggers (dust, animals, hay/flowers, cold air…) were used. Allergic sensitization was defined by at least one positive skin prick test to 12 aeroallergens. Mixture model was used to cluster participants, independently in those without (Asthma-, n = 582) and with asthma (Asthma+, n = 401).

**Results:**

Three clusters were identified in both groups: 1) Cluster A (55% in Asthma-, and 22% in Asthma+) mainly characterized by the absence of nasal symptoms, 2) Cluster B (23% in Asthma-, 36% in Asthma+) mainly characterized by nasal symptoms all over the year, sinusitis and a low prevalence of positive skin prick tests, and 3) Cluster C (22% in Asthma-, 42% in Asthma+) mainly characterized by a peak of nasal symptoms during spring, a high prevalence of positive skin prick tests and a high report of hay fever, allergic rhinitis and conjunctivitis. The highest rate of polysensitization (80%) was found in participants with comorbid asthma and allergic rhinitis.

**Conclusion:**

This cluster analysis highlighted three clusters of rhinitis with similar characteristics than those known by clinicians but differing according to allergic sensitization, and this whatever the asthma status. These clusters could be easily rebuilt using a small number of variables.

## Introduction

Rhinitis is a common respiratory disease worldwide and affects between 20 and 50% of the population depending on the country and on the definition [1–3]. Rhinitis is characterized by nasal congestion, rhinorrhea, itching and/or sneezing [1]. Classically, rhinitis can be divided into two major categories: allergic rhinitis (AR) and non-allergic rhinitis (NAR), with the need of allergic sensitization tests to distinguish between them [1]. Rhinitis is a complex disease, frequently associated with asthma, whatever the allergic sensitization [1] and phenotypes of rhinitis need to be explored.

In a systems biology study (the MeDALL approach, http://medall-fp7.eu/ [4]), classical and novel phenotypes of allergic rhinitis in children ascribed to *hypothesis-driven* and *data-driven* phenotypes were defined using epidemiologic questionnaires [5]. Even if symptoms of rhinitis are similar for children and adults, the disease may differ for comorbidities [6], and till now phenotypes of rhinitis are unexplored in adults.

Unsupervised learning methods (*data driven*) are useful as they allow studying a large data set without historical knowledge, and identifying distinct phenotypes not always detectable by classical approach. On the other hand, these methods can reinforce *hypothesis-driven* approaches and can thus confirm their validity. These methods have already been used with success to identify phenotypes of asthma [7], [8], chronic obstructive pulmonary diseases (COPD) [9], and other respiratory diseases [10]. To our knowledge, only one study has performed cluster analysis in 18 years old participants, all having current rhinitis [11].

The French Epidemiological study of Genetics and Environment of Asthma, bronchial hyperresponsiveness and atopy (EGEA)) is a case-control cohort on asthma. Participants of this study had a very good phenotypic characterization of respiratory health, including allergic sensitization and several specific questions related to rhinitis. The EGEA study offers the unique opportunity to study rhinitis separately in participants with (AS+) and without (AS-) asthma. The objective of this study was to identify distinct types of rhinitis using unsupervised learning methods in adults from the EGEA study.

## Methods

### Study design

EGEA is a French case-control and family study based on an initial group of asthma cases and their first-degree relatives, and a group of controls (EGEA1 [12,13], n = 2047; https://egeanet.vjf.inserm.fr). A first follow-up was conducted between 2003 and 2007.

### Setting

Protocol and descriptive characteristics of the EGEA study have been previously published [12]. Briefly, 2047 children (<16 years) and adult participants were enrolled at baseline, including 348 participants with current asthma from chest clinics, their 1244 first-degree relatives, and 415 population-based controls. Approximately 12 years later, this population was contacted (EGEA2 [14]). Among the alive cohort (n = 2002), 92% (n = 1845) completed a short self-administered questionnaire and among them 1601 had a complete examination. All participants responded to questionnaires based on international standardized tools to diagnose asthma and to determine respiratory and allergic symptoms, treatments, and environmental exposures.

### Participants

**Fig 1:**
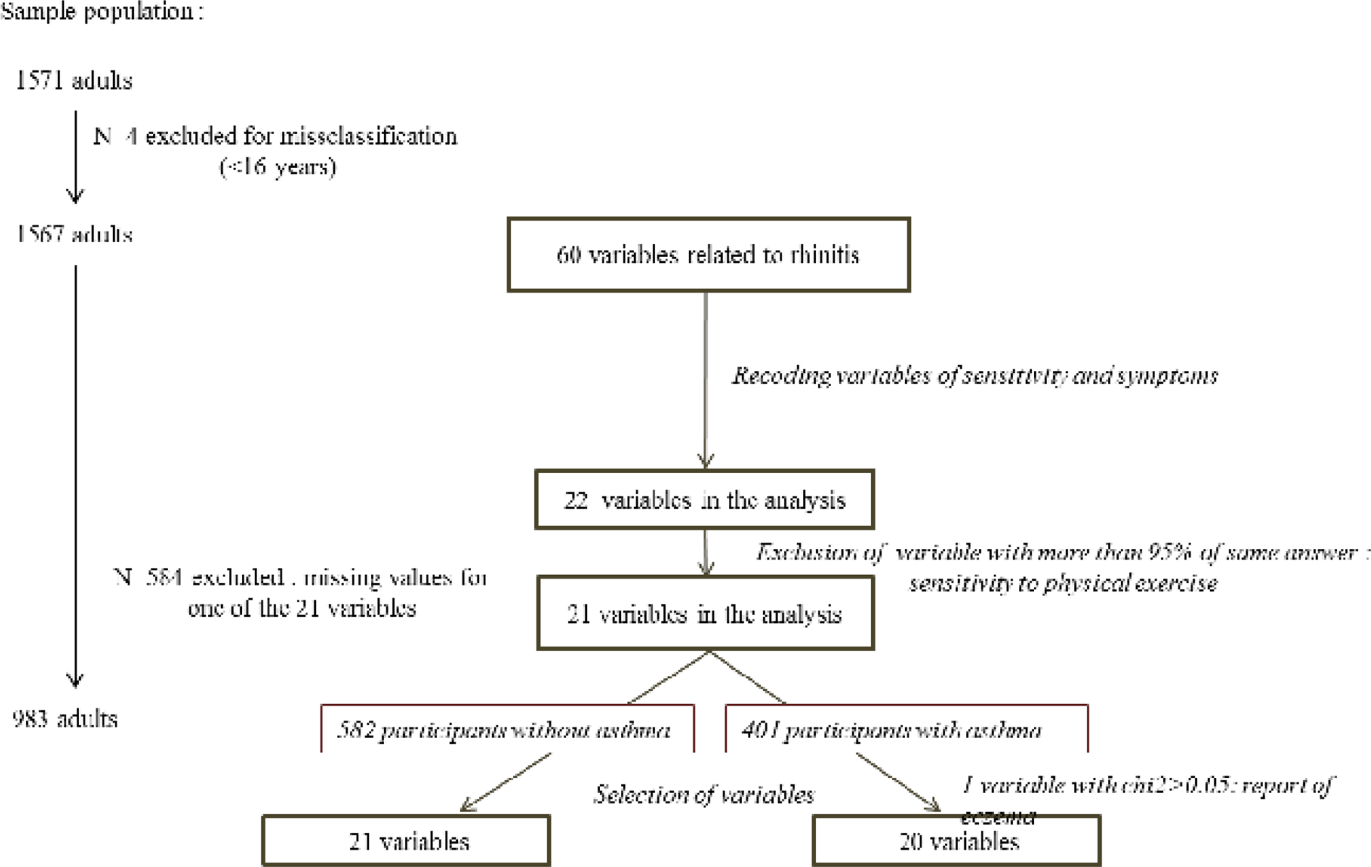
Flow-chart of the variables and of the participants included in the analysis.

The present cross-sectional analysis includes adults at EGEA2 (n = 1571 adults, ≥16 years) without missing data on rhinitis, allergic sensitization and asthma (n = 983, 41% with asthma Fig 1).

### Ethics

Ethical approval was obtained from the relevant institutional review board committees (Cochin Port-Royal Hospital and Necker-Enfants Malades Hospital, Paris). Written informed consent was signed by all participants.

### Variables

#### Rhinitis symptoms

Report of nasal symptoms were defined as a positive answer to *“Have you ever had a problem with sneezing*, *or a runny or a blocked nose when you did not have a cold or the flu?”*. Eyes-associated symptoms were defined as a positive answer to *“Have you ever had itchy or watering eye when you have these nose problems?”*. Current nasal symptoms were defined as a positive answer to the question over the last 12 months. Nasal symptoms were considered as persistent if they occur more than a month per year. They were considered as persistent_low_ if they occur less than 4 days per week and persistent_high_ if they occur more than 4 days per week. Moreover, if the symptoms occurred less than a month per year, persistence of nasal symptoms was considered as intermittent. This classification was built as close as possible to the ARIA guidelines [1], but with some modifications. Answers to the question “*Have these nose problems disturbed you daily activities?*” enabled a score of disturbance from 0 to 3 (0: no, 1: a little bit, 2: moderately, 3: a lot). Participants reported the months in which they had nasal symptoms, and a seasonal pattern was created according to the answer: 0 if no symptom, 1 if symptoms in spring (hay fever), 2 if symptoms in spring/summer, 3 if symptoms in fall/winter, 4 if symptoms all over the year and 5 for the others. Sensitivity to trigger was defined as a positive answer to *“Trigger x usually provoking rhinorrhea”* and *“Trigger x usually provoking sneezing”*. The sensitivity for different triggers was available for animals, weed/flower, dust, cold air, physical exercise, weather, and tobacco smoke exposure (see questionnaires on https://egeanet.vjf.inserm.fr). This sensitivity was coded for the analysis 0: no sensitivity, 1: rhinorrhea or sneezing and 2: rhinorrhea and sneezing. Reports of allergic rhinitis by participants were defined as a positive answer to *“Have you ever had allergic rhinitis?”*, and in the same way for hay fever: *“Have you ever had hay fever?”*. The diagnostic of allergy by a physician was defined as a positive answer to *“Has a doctor ever told you that you are allergic?”*. Positive answers to conjunctivitis, sinusitis and eczema were also considered.

#### Use of medication for rhinitis

Report of use of medication relative to rhinitis were obtained by a positive answer to either: “*Have you took nasal sprays to treat disorders of the nose in the last 12 months?*” or to “*Have you took pills*, *capsules*, *tablets or drugs (other than nasal spray) to treat disorders of the nose in the last 12 months?*”.

#### Asthma

Participants with asthma were defined by a positive answer to either: “*Have you ever had attacks of breathlessness at rest with wheezing?*”, or “*Have you ever had asthma attacks?*”, or if they were recruited as asthmatic cases at the first survey [12].

#### Allergic sensitization

Allergic sensitization was defined by a positive skin prick test (SPT+) with a mean wheal diameter ≥3mm than the negative control for at least one of 12 aeroallergens (indoor: cat, *Dermatophagoides pteronyssinus*, *Blattela germanica*, outdoor: olive, birch, *Parieteria judaica*, timothy grass, *Cupressus and* ragweed pollen, and molds: *Aspergillus*, *Cladosporium herbarum*, *Alternaria tenuis*). Report of allergic immunotherapy since the first survey (EGEA1) was also available.

### Statistical methods

To take into account the specific design of the EGEA study, we conducted the analyses separately in participants without and with asthma.

#### *Hypothesis-driven*: classical phenotypes

The analysis based only on the report of nasal symptoms (yes/no) and allergic sensitization (yes (SPT+)/no) enabled to define four profiles separately for participants with and without asthma: phenotype 1: no nasal symptoms and no allergic sensitization, phenotype 2: allergic sensitization only, phenotype 3: nasal symptoms without allergic sensitization and phenotype 4: nasal symptoms and allergic sensitization. These profiles have already been used to study rhinitis and its relationship with other respiratory diseases [15].

#### *Data-driven*: novel phenotypes

*Data and variable selection*. Sixty variables were first considered, known to be commonly associated with rhinitis or allergic sensitization. After recoding and grouping the variables of the sensibility to different triggers, skin prick test (SPT) and symptoms, 22 variables were available. Sensitivity to “physical exercise” having more than 95% of the same answer was excluded. Twenty-one variables were selected for the analysis: report of nasal symptoms, current/ever symptoms, persistence and disturbance of these symptoms, seasonal pattern, sensitivity to seven triggers, report of allergic rhinitis, hay fever, conjunctivitis, sinusitis and eczema, report of diagnostic of allergy by a physician, SPT, report of spray, report of drug except spray, and allergic immunotherapy since the last survey. A variable selection step (chi2 p-value lower than 0.05) led to select 21 variables for As- and 20 for As+ (Fig 1) and finally the analysis included 983 participants (582 As- and 401 As+) with no missing data (Fig 1 and Table B in S1 Supporting Information).

*Missing Data*. Participants included in the analysis had no missing values, as the data set was built according to that criterion.

*Statistical analysis*. To describe the phenotypes of disease without the need for historical or a priori assumptions, cluster analysis–or clustering- was used [16]. Cluster analysis is a data mining tool for dividing subjects into several groups so that subjects in the same group are more similar (or related) to each other than to those from others groups. This technique defines the distance of each subject from each other based on the combined values-the multidimensional vector- of their measured characteristics.

*Mixture model*. The mixture model is a flexible and powerful parametric algorithm of clustering.where each cluster is mathematically represented by a parametric distribution. The entire data set is then modeled by a mixture of these distributions [17]. The number of clusters associated with the lowest Bayesian Information Criterion (BIC) was chosen. As the solution may depend on the initialization, the algorithm was repeated 100 times and the model with the highest likelihood for mixture model was selected. The χ^2^ test was used to analyze differences between groups for all qualitative variables. ANOVA was used to compare continuous variable according to the group. To display the subjects in two-dimensional space, multiple correspondence analysis was generated from the dataset; each subject was plotted along the two firsts components.

*Tree analysis*. To assess which of the 21 or 20 variables were most predictive of the finale cluster, recursive partitioning based on Classification and Regression Tree (CART) was used. The Gini index was used as the splitting index. The dataset was divided into a training set (70% of the original sample) and a validation set (30% of the original sample) to avoid overfitting. Accuracy was used to select the optimal model using the largest value. The validation of the model on the validation set was assessed using the error-rate value of prediction. Results were expressed as percentage of participants assigned to the right cluster (100%-error rate).

#### Bias

Participants included in the analyses (983) were not significantly different of those not included in the analyses (n = 588, see Table A in S1 Supporting Information) neither for age, sex, body mass index (BMI), nor for nasal symptoms, allergic sensitization, lung function and asthma status.

Due to the familial design of the study, a sensitivity analysis was conducted in a sub-sample of the population with one randomly selected member per family (n = 684 participants, 420 without asthma and 264 with asthma).

All the analyses were performed using the R statistical software. The Rmixmod package (http://cran.r-project.org/web/packages/Rmixmod/index.html) was used to run the algorithm of mixture models, and the rpart package was used to perform the tree analysis.

## Results

The characteristics of the 983 adults according to their asthma status are summarized in Table 1. Participants with asthma had significantly lower Forced Expiratory Volume in one second (FEV_1_) level, more often bronchial hyper-responsiveness (BHR), allergic sensitization (SPT+), and reported more often nasal symptoms, AR and hay fever than participants without asthma.

**Table 1:** Characteristics of adult participants.

		All (n = 983)	Participants without asthma (n = 582)	Participants with asthma (n = 401)	P value*
**Age, mean ± sd**		42.6 ± 16.5	45.9 ± 15.9	37.7 ± 16.1	<0.001
**Sex, women %**		49.5	51.6	46.6	0.13
**Tobacco status, %**	**Non-smoker **	49.6	48.0	51.9	0.052
	**Ex-smoker**	26.3	29.1	22.2	
	**Smoker**	24.1	22.9	25.9	
**BMI(kg/m2), %**	**<20**	10.7	9.6	12.2	0.09
	**[20–25]**	49.6	48.5	51.4	
	**[25–30]**	29.4	32.3	25.2	
	**> = 30**	10.3	9.6	11.2	
**Educational level, %**	** Low**	24.5	28.7	18.5	<0.001
	**Med**	27.7	25.1	31.5	
	**high**	47.8	46.2	50.0	
**Allergic sensitization, %**		65.2	46.8	82.0	<0.001
Ever asthma, %		40.8	-	-	
**BHR** ^#^ **, n**		663	396	n = 267	
**%**		44.3	27.0	70.0	<0.001
**FEV**_**1**_ **% predicted, mean±sd**		102 ± 18	106 ± 16	97 ± 18	<0.001
**Nasal symptoms, %**		58.9	45.5	78.3	<0.001
**Reports of AR, %**		36.2	21.8	57.1	<0.001
**Reports of Hay fever, %**		38.8	24.7	59.1	<0.001

BMI = Body Mass Index, FEV1 = Forced Expiratory Volume, AR: allergic rhinitis

#: BHR: Bronchial Hyper Responsiveness (Methacholine test, PD20≤4 mg, Methacholine challenge test was not performed if baseline FEV1 <80% predicted, PD20 = Provocative Dose). BHR was then available for 663 participants (396 without asthma and 267 with asthma).

SPT+: a mean wheal diameter ≥3mm than the negative control for at least one of 12 aeroallergens.

* comparing participants without and with asthma (χ_2_ test)

### *Hypothesis-driven* (classical phenotypes) (Table C and D in S1 Supporting Information)

Varied prevalence of the four phenotypes were observed according to the asthma status: Phenotype 1: no symptoms, no allergic sensitization (39% for As- and 4% for As+), phenotype 2: allergic sensitization only (15 for As-and 17% for As+), phenotype 3: nasal symptoms without allergic sensitization (24 for As- and 14% for As+), and phenotype 4: nasal symptoms and allergic sensitization (22 for As- and 65% for As+). Whatever the asthma status, participants of phenotype 4 had the highest rates of hay fever report, allergic conjunctivitis report and sensitivity to hay/flower and animals. Participants of phenotype 3 had the highest rates of sinusitis report and of sensitivity to cold air.

### *Data-driven* (novel phenotypes obtained by cluster analysis)

**Fig 2:**
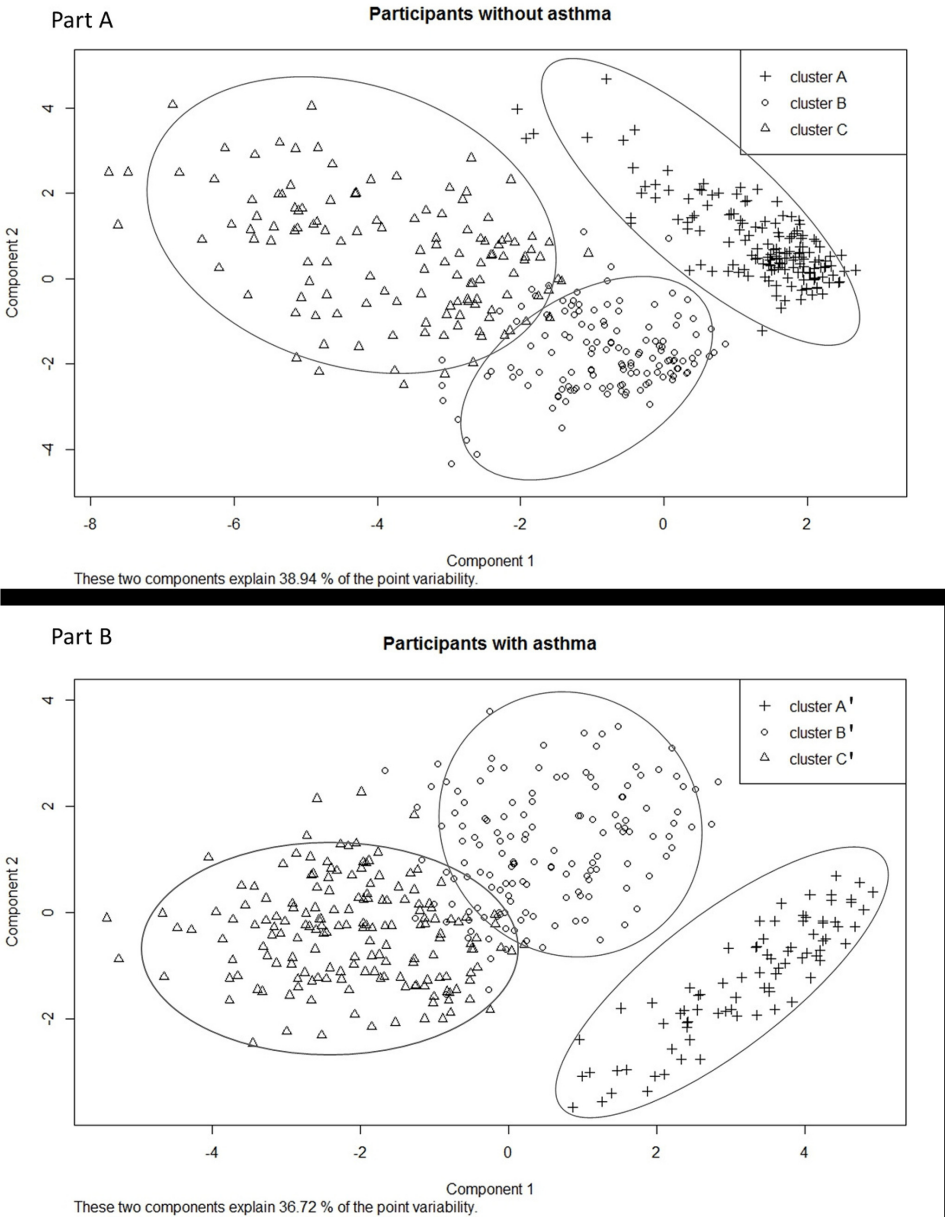
Visualization of the clusters for participants without asthma (Part A) and participants with asthma (Part B) on the first factorial map.

A three-cluster model was selected as the best model for both As- and As+ participants using the BIC criterion (S1 Fig) and the three clusters were well separated (Fig 2) whatever the asthma status. In participants without asthma: 55% of the participants were in cluster A, 23% in cluster B (23%) and 22% in cluster C. In participants with asthma: 22% of the participants were in the cluster A’, 36% in the cluster B’ and 42% in cluster C’.

Whatever the asthma status, cluster A and A’ were characterized by the absence of nasal symptoms, low reports of AR, hay fever and sensitivity to all triggers (Tables 2 and 3).

**Table 2:** Characteristics of participants without asthma according to each cluster.

		Cluster A-no rhinitis- (n = 317)	Cluster B-non-allergic rhinitis- (n = 136)	Cluster C-allergic rhinitis- (n = 129)	p-value*
**Age, mean ± sd**		46.7 ± 16.2	48.9 ± 15.5	40.9 ± 14.5	<0.001
**Sex, women %**		46.7	58.1	56.6	0.036
**Tobacco, %**	**Non-smoker**	47.2	47.0	51.2	0.90
** **	**Ex-smoker**	30.0	30.2	25.6	
** **	**Smoker**	22.8	22.88	23.3	
**BMI (kg/m2), %**	**<20**	10.1	8.8	9.3	0.53
** **	**[20–25]**	47.0	47.8	52.7	
** **	**[25–30]**	33.4	36.0	25.6	
** **	**> = 30**	9.5	7.4	12.4	
**Educational level, %**	**Low**	31.2	34.6	16.3	0.007
** **	**Medium**	25.2	23.5	26.4	
** **	**High**	43.5	41.9	57.4	
**Nasal symptoms, %**	**No symptoms**	100.0	0.0	0.0	<0.001
	**Symptoms without eyes symptoms**	0.0	71.3	17.1	
** **	**Symptoms with eyes symptoms**	0.0	28.7	82.9	
**Type of nasal symptoms, %**	**No symptoms**	100	0.0	0.0	-
	**Symptoms: ever but not current**	0.0	4.4	0.0	
** **	**Ever and current symptoms**	0.0	95.6	100	
**Persistence of nasal symptoms, %**	**Intermittent**	-	50.0	40.3	0.22
	**Persistent**_**low**_	-	18.4	25.6	
	**Persistent**_**high**_	-	31.6	34.1	
**Disturbance due to nasal**	**No**	-	77.2	42.6	<0.001
**symptoms, %**	**Low**	-	17.7	39.5	
	**Medium**	-	4.4	14	
	**High**	-	0.7	3.90	
**Allergic sensitization,%**	SPT = 0	71.9	80.9	23.3	<0.001
	SPT = 1	17	10.3	23.3	
	SPT = 2	7.6	4.4	19.4	
	SPT>2	3.5	4.4	34.1	
**Report of diagnosis of allergy by a physician, %**		15.5	18.4	72.1	<0.001
**Immunotherapy since first survey (EGEA1)**		1.60	0.0	13.2	<0.001
**Age of onset of nasal symptoms, mean ± sd**	**(n = 224)**	-	33.7 ± 18.2	22.1 ± 14.1	<0.001
**Report of allergic rhinitis, %**		5.70	6.6	77.5	<0.001
**Report of hay fever, %**		10.7	10.3	74.4	<0.001
**Report of conjunctivitis, %**		13.4	22.1	49.6	<0.001
**Report of sinusitis, %**		34.7	55.1	56.6	<0.001
**Report of eczema, %**		22.1	30.9	36.4	0.005
**Sensitivity to hay/flowers, %**	**No sensitivity**	89.3	82.4	29.5	<0.0001
	**Rhinorrhea or sneezing**	8.2	16.9	30.2	
	**Rhinorrhea and sneezing**	2.5	0.7	40.3	
**Sensitivity to animals, %**	**No sensitivity**	98.1	100	78.3	<0.0001
	**Rhinorrhea or sneezing**	1.60	0.0	13.2	
	**Rhinorrhea and sneezing**	0.30	0.0	8.50	
**Sensitivity to dust, %**	**No sensitivity**	74.1	58.1	27.1	<0.0001
	**Rhinorrhea or sneezing**	24.3	39.7	46.5	
** **	**Rhinorrhea and sneezing**	1.60	2.20	26.4	
**Sensitivity to tobacco smoke, %**	**No sensitivity**	98.1	90.4	87.5	<0.0001
	**Rhinorrhea or sneezing**	1.60	7.40	11.7	
** **	**Rhinorrhea and sneezing**	0.30	2.20	0.80	
**Sensitivity to cold air, %**	**No sensitivity**	84.2	66.9	67.4	<0.0001
	**Rhinorrhea or sneezing**	15.1	30.2	27.9	
** **	**Rhinorrhea and sneezing**	0.60	2.90	4.70	
**Sensitivity to weather, %**	**No sensitivity**	97.5	88.2	83.0	<0.0001
	**Rhinorrhea or sneezing**	1.60	11.8	10.8	
** **	**Rhinorrhea and sneezing**	0.90	0.00	6.20	
**Use of nasal spray in the last 12 months, %**		23.0	39.7	54.3	<0.0001
**Use of other drug in the last 12 months, %**		17.7	27.9	62.0	<0.0001

BMI = Body Mass Index

*p-value overall

**Table 3:** Characteristics of participants with asthma according to each cluster.

		Cluster A’-no rhinitis- (n = 87)	Cluster B’-non-allergic rhinitis- (n = 144)	Cluster C’-allergic rhinitis-(n = 170)	p-value*
**Age, mean ± sd**		39.9 ± 16.8	38.5 ± 17.6	35.9 ± 14.3	<0.001
**Sex, women %**	** **	44.8	44.4	49.4	0.63
**Tobacco, %**	**Non smoker**	51.7	45.1	57.7	0.054
** **	**Ex-smoker**	28.7	25.0	16.5	
** **	**Smoker**	19.5	29.9	25.9	
**BMI (kg/m2), %**	**<20**	8.1	11.1	15.3	0.32
** **	**[20–25]**	52.9	47.2	54.1	
** **	**[25–30]**	25.3	28.5	22.4	
** **	**> = 30**	13.8	13.2	8.2	
**Educational level, %**	**Low**	25.3	20.8	13.0	0.12
** **	**Medium**	31.0	31.9	32.4	
** **	**High**	43.7	47.2	55.6	
**Nasal symptoms,**	**No symptoms**	100	0.0	0.0	<0.001
**%**	**Symptoms without eyes symptoms**	0.0	43.8	7.6	
** **	**Symptoms with eyes symptoms**	0.0	56.2	92.4	
**Type of nasal symptoms**	**No symptoms**	100	0.0	0.0	<0.001
**%**	**Symptoms: ever but not current**	0.0	1.4	0.0	
** **	**Ever and current symptoms**	0.0	98.6	100	
**Persistence of nasal symptoms, %**	**Intermittent**	-	50.0	20.6	<0.001
	**Persistent**_**low**_	-	29.2	31.2	
	**Persistent**_**high**_	-	20.8	48.2	
**Allergic sensitization,%**	**SPT = 0**	19.5	33.3	4.1	<0.001
	**SPT = 1**	27.6	19.4	15.3	
	**SPT = 2**	20.7	14.6	19.4	
	**SPT>2**	32.2	32.6	61.2	
**Report of diagnosis of allergy by a physician, %**		58.6	60.4	96.5	<0.001
**Immunotherapy since first survey (EGEA1)**		8.0	8.3	22.9	<0.001
**Age of onset of nasal symptoms, mean ± sd**	**(n = 290)**	-	19.3 ± 86.9	11.5 ± 10.0	<0.001
**Report of allergic rhinitis, %**		26.4	41.7	85.9		<0.001
**Report of hay fever, %**		35.6	34.0	92.4		<0.001
**Report of conjunctivitis, %**		26.4	30.6	69.4		<0.001
**Report of sinusitis, %**		46.0	49.3	60.0		0.053
**Report of eczema, %**		42.5	47.2	53.5		0.22
**Sensitivity to hay/flowers, %**	**No sensitivity**	77.0	76.4	10.0	<0.0001
	**Rhinorrhea or sneezing**	12.6	20.1	30.0	
** **	**Rhinorrhea and sneezing**	10.3	3.5	60.0	
**Sensitivity to animals, %**	**No sensitivity**	88.5	81.3	52.9	<0.0001
	**Rhinorrhea or sneezing**	6.9	12.5	18.8	
** **	**Rhinorrhea and sneezing**	4.6	6.3	28.2	
**Sensitivity to dust, %**	**No sensitivity**	64.4	50.7	16.5	<0.0001
	**Rhinorrhea or sneezing**	25.3	37.5	42.9	
** **	**Rhinorrhea and sneezing**	10.3	11.8	40.6	
**Sensitivity to tobacco smoke, %**	**No sensitivity**	95.4	90.2	78.2	0.0002
	**Rhinorrhea or sneezing**	1.0	9.1	14.1	
** **	**Rhinorrhea and sneezing**	0.0	0.7	4.7	
**Sensitivity to cold air, %**	**No sensitivity**	86.2	71.5	63.5	0.001
	**Rhinorrhea or sneezing**	13.8	26.4	30	
** **	**Rhinorrhea and sneezing**	0.0	2.1	6.5	
**Sensitivity to weather, %**	**No sensitivity**	94.3	86.8	70.6	<0.0001
	**Rhinorrhea or sneezing**	5.8	9.7	14.7	
	**Rhinorrhea and sneezing**	0.0	3.5	14.7	
**Disturbance due to nasal symptoms, %**	**No**	-	65.3	29.4	<0.001
	**Low**	-	20.1	34.1	
	**Medium**	-	11.1	23.5	
	**High**	-	3.5	12.9	
**Use of nasal spray in the last 12 months, %**		44.8	47.2	64.1		0.0018
**Use of other drug in the last 12 months, %**		42.5	42.4	80.6		<0,0001
**Age of onset of asthma, mean ± sd**		16,8±16,2	15,6±15,7	12,2±13,2		0.6 (A vs B) and 0.02 (A vs C)
**BHR, %**	**(n = 267)**	64.4	75.0	69.0		0.36
**FEV**_**1**_ **% predicted, mean±sd**		96 ± 0.18	98 ± 0.16	96 ± 0.22		0.42

BMI = Body Mass Index, FEV1 = Forced Expiratory Volume, #: BHR: Bronchial Hyper Responsiveness (Methacholine test, PD20≤4 mg, Methacholine challenge test was not performed if baseline FEV1 <80% predicted, PD20 = Provocative Dose). BHR was then available for 663 participants (396 without asthma and 267 with asthma).

*p-value overall

#### In participants without asthma (Table 2)

**Cluster B** was characterized by the highest rate of nasal symptoms without eyes-symptoms associated, a high report of sinusitis and eczema, and a low report of AR, hay fever and conjunctivitis as compared to cluster A. The rate of allergic sensitization was lower than for cluster A. Sensitivities to different triggers were lower for hay/flower and animals but higher for cold air, compared to cluster A (Table 2). **Cluster C** was characterized by the highest rate of nasal symptom mostly associated with eyes-symptoms, the highest rate of SPT, the highest rate of sinusitis, eczema and conjunctivitis reports and the highest rates of sensitivity to hay/flower, animals, dust and weather.

The allergic sensitization was mostly monosensitization for clusters A and B while it was mostly polysensitization for cluster C (Table 2). Among participants with allergic sensitization (SPT+), 61% of cluster A, 54% of cluster B, and 30% of cluster C were monosensitized, mostly for *Dermatophagoides pteronyssinus*.

Regarding seasonality of symptoms, cluster B reported symptoms all over the year whereas cluster C reported symptoms mainly during spring (hay/flower season). The score of disturbance was higher for cluster C than for cluster B. No significant difference between clusters was observed in term of persistent or intermittent symptoms.

#### In participants with asthma (Table 3)

**Cluster B’** was characterized by a high rate of nasal symptoms, a low report of AR, hay fever and conjunctivitis and the lowest rate of SPT. Sensitivities to different triggers were low for hay/flower and animals but high for cold air, tobacco and weather. **Cluster C’** was characterized by the highest rate of nasal symptoms with eyes-symptoms, the highest rate of allergic sensitization, the highest rates of report of AR, hay fever, sinusitis and conjunctivitis and the highest rates of sensitivity to hay/flower, animals, dust and weather.

The allergic sensitization rate was high whatever the cluster (Table 3), and mostly characterized by a polysensitization. Similarly to participants without asthma, monosensitization was higher for clusters A’ and B’. Among participants with allergic sensitization (SPT+), 34% of cluster A’, 29% of cluster B’, and 16% of cluster C’ were monosensitized mostly for *Dermatophagoides pteronyssinus*. The polysensitization rate was particularly high in cluster C’ (80%).

Cluster B’ and cluster C’ reported symptoms all over the year but cluster C’ had a very high peak during spring. The score of disturbance due to nasal symptoms was higher for cluster C’ than for cluster B’. Cluster C’ declared more persistent_high_ than persistent_low_ symptoms while cluster B’ declared more intermittent or persistent_low_ symptoms. The age of onset of asthma was lower for participants of cluster C’ than for cluster A’ and B’. BHR was higher in participants of cluster B’ than in participants of clusters A’ or C’, but the difference was not significant.

**Whatever the asthma status,** the report of spray or pills/tablet use to nasal problem was higher for cluster C (respectively C’) than for cluster B (resp. B’). Participants of cluster B (resp. B’) reported more use of spray than pills/tablet whereas participants of cluster C (resp. C’) reported more use of pills/tablet than spray. Age of onset of nasal symptoms was lower in participants with asthma than in those without asthma, and whatever the asthma status, participants of cluster C (resp. C’) had an age of onset of nasal symptoms significantly lower than participants of cluster B (resp. B’).

### Comparison between *data-driven* clusters and *hypothesis-driven* phenotypes (Tables 4 and 5)

**Table 4:** Comparison of the repartition of the participants without asthma into the different *hypothesis-driven*’s phenotypes and *data-driven*’s cluster.

		*Data-driven* clusters			
	n (%)	A (No rhinitis)	B (NAR)	C (AR)	
*Hypothesis-driven* Phenotypes	1 (no symptoms, no SPT)	228 (39)	0	0	228
	2 (no symptoms, SPT+)	89 (15)	0	0	89
	3 (symptoms, no SPT ~ NAR)	0	110 (19)	**30 (5)**	140
	4 (symptoms, SPT+ ~AR)	0	**26 (5)**	99 (17)	125
		317	136	129	**582**

**Table 5:** Comparison of the repartition of the participants with asthma into the different *hypothesis-driven*’s phenotypes and *data-driven*’s cluster.

		*Data-driven* clusters			
	n (%)	A’ (No rhinitis)	B’ (NAR)	C’ (AR)	
*Hypothesis-driven* Phenotypes	1 (no symptoms, no SPT)	17 (4)	0	0	17
	2 (no symptoms, SPT+)	70 (17)	0	0	70
	3 (symptoms, no SPT ~ NAR)	0	48 (12)	**7 (2)**	55
	4 (symptoms, SPT+ ~AR)	0	**96 (24)**	163 (41)	259
		87	144	170	**401**

Clusters obtained by *data-driven* approach may be easily assimilated to no rhinitis (NoR: cluster A and A’), non-allergic rhinitis (NAR: cluster B and B’) and allergic rhinitis (AR: cluster C and C’) based on their characteristics. These clusters are similar to the phenotypes 1, 3 and 4 from the classical *hypothesis-driven* phenotypes a *prima facie* but differ in their internal characteristics and particularly regarding the allergic sensitization. When comparing *data-driven* and *hypothesis-driven* approach, 10% of participants without asthma were not classified in the same category by the two approaches and 26% of participants with asthma. Considering only participants having nasal symptoms, 21% of participants without asthma were not classified in the same category by the two approaches and 30% of participants with asthma.

### Decision tree

**Fig 3:**
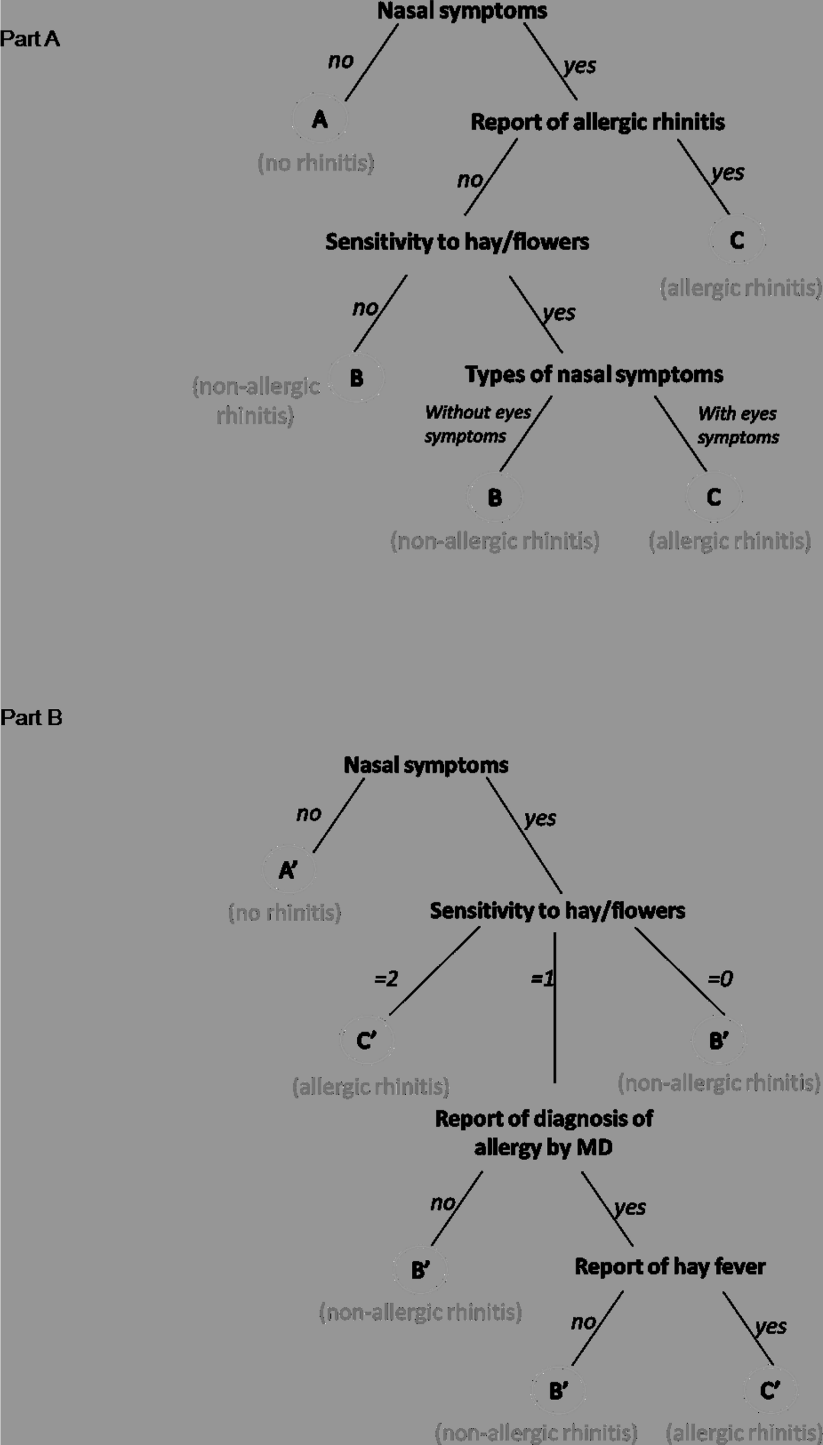
Classification tree obtained with the most predictive variables in participants without (Part A) and with asthma (Part B).

For participants without asthma, a classification tree on the 21 variables enabled to highlight 4 variables as being the most important to discriminate the cluster and particularly to distinguish cluster B from cluster C: report of nasal symptoms, report of AR, sensitivity to hay/flowers stimuli and type of nasal symptoms-with or without eyes symptoms- (Fig 3, Part A). Using only these 4 variables, 96% of the participants were assigned to the correct cluster.

For participants with asthma, a classification tree on the 20 variables enabled to highlight 4 variables as being the most important to discriminate the cluster and particularly to distinguish cluster B’ from cluster C’: report of nasal symptoms, sensitivity to “hay/flower” stimuli, diagnosis of allergy by a MD and report of hay fever (Fig 3, Part B). Using only these 4 variables, 87% of the participants were assigned to the correct cluster.

### Sensitivity analysis

The cluster analysis on the sub-sample of the population including only one member per family has shown very similar results than the study on the 983 participants (same number of cluster, same characteristics–data not shown-).

## Discussion

This study using a clustering approach identified three rhinitis phenotypes in adults with almost no overlap between them. They are similar to the *hypothesis-driven* phenotypes of no rhinitis (NoR: cluster A and A’), non-allergic rhinitis (NAR: cluster B and B’) and allergic rhinitis (AR: cluster C and C’). However, hypothesis and data-driven phenotypes differ in terms of allergic sensitization. Near of a quarter of participants without asthma would have been considered as having allergic rhinitis considering the *hypothesis-driven* phenotypes whereas they have a non-allergic rhinitis pattern. Our study was able to highlight the importance of the NAR phenotypes, less understood and which need to be studied [18] and enhanced the importance of the non-allergic component in rhinitis. In participants with asthma, the AR cluster was associated with the highest rate of allergic sensitization and number of allergens, suggesting a comorbid effect of asthma and allergic rhinitis on the polysensitization.

Participants of the study had a very good phenotypic characterization of respiratory health and allergic sensitization that gave us the opportunity to consider several questions related to rhinitis. The design of the study allowed us to compare the characteristics of rhinitis phenotypes according to the asthma status. One of the limitations is that the sample is not big enough to study finest clusters and particularly mixed rhinitis (participants having non allergic and allergic rhinitis). Our analysis did not identify some very specific adult rhinitis phenotypes such as hormonal rhinitis [19] probably because of their low prevalence. Overall, to our knowledge, our study is the first with such detailed questionnaires and allergic sensitization available. As the analyses were performed separately for participants without and with asthma, our results cannot be transposed to population-based studies.

Cluster of rhinitis have consistent characteristics with previous literature and clinician’s knowledge. We reported that AR cluster was more related to conjunctivitis and eyes-associated symptoms whereas NAR cluster was more related to sinusitis. NAR cluster was more associated with sensitivity to trigger as cold air whereas AR cluster was more associated with sensitivity to multiple allergens as pet, hay, and flower. Age at onset of nasal symptoms was lower for AR cluster than for NAR cluster. These results are concordant with several papers comparing allergic rhinitis to non-allergic rhinitis [20], [21], [22]. Overall, it is reassuring that *prima facie*, unsupervised approaches find similar phenotypes than the ones used in the clinical setting.

Interestingly, we observed that AR cluster was associated with more severe symptoms (greater disturbance) than NAR cluster. This result is consistent with the studies by Bachert [23] and Di Lorenzo [21] but discordant with the study by Molgaard [24]. This discordance between studies seems not to be due to the design of the studies, but to the difference in the definitions of the types of rhinitis and particularly in the way that allergic and non-allergic rhinitis were differentiated. Overall, the definitions and particularly the way to define the allergic part of rhinitis seem to be crucial to establish the characteristics of the phenotypes. Furthermore, whatever the cluster of rhinitis (NAR or AR), we found that almost all of the participants who reported ever rhinitis also reported current nasal symptoms which suggest that considering rhinitis ever or current rhinitis would give the same result.

Prevalence and repartition of non-allergic and allergic rhinitis are very different according to the study: between 63% and 77% of rhinitis would be of allergic type [22,24], but some other studies argue that over 75% of rhinitis is non-allergic rhinitis or mixed rhinitis [20]. In our study, we found a higher prevalence of rhinitis in participants with asthma. However, within each asthma status, the prevalence of NAR cluster was similar to that of AR cluster.

Whereas rhinitis is classically divided in allergic and non-allergic rhinitis based on the allergic sensitization, our results suggest that allergic sensitization may be insufficient to differentiate correctly AR and NAR and to make the diagnosis of AR. This result is concordant with a paper studying predictor factors to differentiate between allergic and non-allergic rhinitis in children [25], which found out that features of rhinitis as seasonality, moderate/severe symptoms help in the differentiation of rhinitis. Di Lorenzo [21] has showed that several clinical and laboratory parameters may help to reinforce or exclude the diagnosis of AR obtained with SPT, and Quillen said that: “allergy testing is not necessary in all patients but may be useful in ambiguous or complicated cases”[26]. Finally, Berstein [27] said that “taking into account age of symptom onset, family history, quantification of inciting allergic and/or non-allergic triggers, and seasonality followed by aeroallergen skin testing to assess atopic status has been shown to be the most useful approach for clearly differentiate rhinitis subtypes”. Overall, these results are concordant with known complexity to define phenotypes of rhinitis.

This study enabled to validate and confirm phenotypes of rhinitis often described in the literature, but for the first time highlighted in a statistical way. Thanks to a classification tree, our results showed the clinical interest of using only a few numbers of questions to classify the participants in the 3 clusters and particularly to distinguish between non-allergic and allergic rhinitis. These questions are often available in respiratory epidemiological study making easier the reconstruction and the use by general physician and pharmacist.

In conclusion, taking into account all available specific questions related to rhinitis, a cluster analysis enabled to highlight three clusters of rhinitis with similar characteristics than those known by clinicians but differing according to allergic sensitization, and this whatever the asthma status. The clusters obtained by *data-driven* approach may be considered as “smoothed” phenotypes compared to the ones obtained only using nasal symptoms and allergic sensitization. These clusters could now be used to study the association with biological and environmental factors. Overall, although cluster analysis is thought to be hypothesis generating, studies in asthma, COPD and now rhinitis show that is may also be useful in hypothesis confirmation.

## Supporting Information

S1 Fig BIC criterion according to the number of cluster for participants without (Part A) and with (Part B) asthma. (EPS)

S1 Supporting Information Comparison of the characteristics of the participants included and non-included in the analysis (Table A). Missing values for each variables (Table B). Description of the participants without asthma according to the four classical phenotypes (hypothesis driven) (Table C). Description of the participants with asthma according to the four classical phenotypes (hypothesis driven) (Table D).
(DOC)
