# Supplementary material for: Characterization of Rhinitis According to the Asthma Status in Adults Using an Unsupervised Approach in the EGEA Study
Source: PLoS One. 2015 Aug 26;10(8):e0136191. doi: 10.1371/journal.pone.0136191 (PMC4550236; doi:10.1371/journal.pone.0136191)
Supplement: S1 Supporting Information — Missing values for each variables (Table B). Description of the participants without asthma according to the four classical phenotypes (hypothesis driven) (Table C). Description of the participants with asthma according to the four classical phenotypes (hypothesis driven) (Table D). (DOC) [file pone.0136191.s002.doc]

**Characterization of rhinitis according to the asthma status in adults using an unsupervised approach in the EGEA study**

# Emilie Burte^1,2,*^, Jean Bousquet,^1,2,3^, Raphaëlle Varraso,^1,2^, Frédéric Gormand,^4^, Jocelyne Just,^5,6^, Régis Matran,^7^, Isabelle Pin,^8,9,10,11^, Valérie Siroux,^8,9,10^, Bénédicte Jacquemin,^1,2,12,#^, Rachel Nadif,^1,2,$#^

1. INSERM, U1168, VIMA: Aging and chronic diseases. Epidemiological and Public health approaches, F-94807, Villejuif, France

2. Univ Versailles St-Quentin-en-Yvelines, UMR-S 1168, F-78180, Montigny le Bretonneux, France

3 University hospital, Montpellier, France 4. CHU de Lyon, Pneumology Department, Lyon, France

5. Allergology Department, Centre de l’Asthme et des Allergies. Hôpital Armand-Trousseau (APHP), APHP, Paris, France

6. Université Paris 6 Pierre et Marie Curie, Paris, France

7. Univ Lille Nord de France, F-59000, Lille, France

8. INSERM, IAB, Team of Environmental Epidemiology applied to Reproduction and Respiratory Health, F-38000 Grenoble, France.

9. Univ. Grenoble Alpes, F-38000 Grenoble, France.

10. CHU de Grenoble, F-38000 Grenoble, France.

11. CHU de Grenoble, Pediatric Department, F-38000, Grenoble, France

12. CREAL-Centre for Research in Environmental Epidemiology Parc de Recerca Biomèdica de Barcelona, Barcelona, Spain.

### * Corresponding author

E-mail: [emilie.burte@inserm.fr](mailto:rachel.nadif@inserm.fr) (EB)

**^#^** These authors contributed equally to this work

**Methods**

A lung function test with methacholine challenge was performed using a standardized protocol with similar equipment across centers according to the ATS/ERS guidelines (S1). Methacholine challenge was performed unless baseline FEV1 <80% predicted.

**References**

S1. Miller MR, Hankinson J, Brusasco V, Burgos F, Casaburi R, Coates a, et al. Standardisation of spirometry. Eur Respir J. 2005 Aug;26(2):319–38.

Table A. Comparison of the characteristics of the participants included and non-included in the analysis

|  | | **Not included (n=588)**  **(n)=** | **Included (n=983)** | **p-value** |
| --- | --- | --- | --- | --- |
| age, mean± sd | | 43.1±16.6 | 42.6±16.5 | 0.52 |
| Sex, women % | | 52.4 | 49.5 | 0.28 |
| Tobacco status, % | Non smoker | 50.4 | 49.6 | 0.40 |
|  | Ex smoker | 28.2 | 26.3 |  |
|  | Smoker | 21.3 | 24.1 |  |
| BMI, % | <20 | 10.8 | 10.7 | 0.09 |
|  | [20-25] | 56 | 49.6 |  |
|  | [25-30] | 23 | 29.4 |  |
|  | >=30 | 10.2 | 10.3 |  |
| SPT | SPT=0 | 40.9 | 44.8 | 0.61 |
|  | SPT=1 | 20.3 | 17.9 |  |
|  | SPT=2 | 12.8 | 12.9 |  |
|  | SPT>2 | 25.9 | 24.4 |  |
| Ever asthma, % | | 40.8 | 40.8 | 0.99 |
| FEV1, % predict | | 102.5±0.19 | 102.4±0.18 | 0.89 |
| BHR, % | | 42.9 (n=203) | 44.3 (n=663) | 0.71 |
| Report of nasal symptoms, % | | 63.3 | 58.9 | 0.09 |
| Reports of AR, % | | 33.33 | 36.22 | 0.26 |
| Reports of hay fever, % | | 35 | 38.76 | 0.14 |
| Educational level, % | Low | 24.6 | 24.5 | 0.94 |
|  | Med | 26.9 | 27.7 |  |
|  | high | 48.5 | 47.8 |  |

BMI= Body Mass Index, SPT: Skin Prick Test, BHR: Bronchial HyperResponsiveness (Methacholine test, PD20≤4 mg ), FEV1= Forced Expiratory Volume in 1s , AR: allergic rhinitis

Table B. Missing values for each variables

|  | **Variable** | **Missing (N=)** |
| --- | --- | --- |
| Nasal symptoms | Type (associated with eyes symptoms or not) | 6 |
|  | Current or ever | 12 |
|  | Persistence | 103 |
|  | Disturbance | 103 |
|  | Month profile | 127 |
| Report of other related disease | Report of allergic rhinitis | 33 |
|  | Report of hay fever | 25 |
|  | Report of conjunctivitis | 30 |
|  | Report of sinusitis | 9 |
|  | Report of eczema | 10 |
| Sensitivity to stimuli | Animals | 64 |
|  | Hay/flowers | 59 |
|  | Tobacco | 2 |
|  | Cold air | 53 |
|  | Effort | 74 |
|  | Dust | 58 |
|  | Weather | 192 |
| Drug consumption | Spray for nasal problem , last 12 month | 13 |
|  | Other drugs for nasal problem(not spray), last 12 months | 107 |
| Report | Desensitization since first survey (EGEA1) | 89 |
|  | Diagnosis of allergy (by a physician) | 86 |
| Allergic sensitization | SPT+ | 266 |
| Asthma status |  | 0 |

Table C. Description of the participants without asthma according to the four classical phenotypes (hypothesis driven)

|  |  | **Phenotype 1: No symptoms, no SPT (n=228)** | **Phenotype 2: SPT only(n=89)** | **Phenotype 3: symptoms, no SPT(n=140)** | **Phenotype 4: Symptoms and SPT (n=125)** |
| --- | --- | --- | --- | --- | --- |
| **age, mean± sd** | | 48.3±15.9 | 42.7±16.6 | 50.6±14.8 | 38.7±13.8 |
| **Sex, women %** | | 49.1 | 40,5 | 61,4 | 52.8 |
| **Tobacco status, %** | **Non-smoker** | 45.8 | 50.6 | 47.9 | 50.4 |
|  | **Ex-smoker** | 32.6 | 23.6 | 33.6 | 21.6 |
|  | **Smoker** | 21.6 | 25.8 | 18.6 | 28 |
| **BMI, %** | **<20** | 9.2 | 12.4 | 5.7 | 12.8 |
|  | **[20-25]** | 46.5 | 48.3 | 47.1 | 53.6 |
|  | **[25-30]** | 32.9 | 34.8 | 36.4 | 24.8 |
|  | **>=30** | 11.4 | 4.5 | 10.7 | 8.8 |
| **Nasal symptoms, %** | **Symptoms without eye symptoms** |  |  | 62.9 | 24.8 |
|  | **Symptoms with eye symptoms** |  |  | 37.1 | 75.2 |
| **Type of nasal symptoms, %** | **ever but not current** |  |  | 2.9 | 1.6 |
|  | **ever and current** |  |  | 97.1 | 98.4 |
| **Report of allergic rhinitis*, %** | | 5.3 | 6.7 | 25.7 | 58.4 |
| **Report of hay fever*, %** | | 6.6 | 21.4 | 17.9 | 68 |
| **Report of conjonctivitis*, %** | | 12.3 | 16.9 | 27.1 | 44.8 |
| **Report of sinusitis*, %** | | 35.1 | 33.7 | 58.6 | 52.8 |
| **Report of eczema*, %** | | 17.5 | 33.7 | 35.7 | 31.2 |
| **Diagnostic of allergy*, %** | | 13.2 | 21.4 | 30.7 | 60.0 |
| **Sensitivity to hay/flowers, %** | **No sensitivity** | 92.5 | 80.9 | 76.4 | 34.4 |
|  | **Rhinorrhea or sneezing** | 6.1 | 13.5 | 18.6 | 28.8 |
|  | **Rhinorrhea and sneezing** | 1.3 | 5.6 | 5 | 36.8 |
| **Sensitivity to animals, %** | **No sensitivity** | 98.3 | 97.8 | 99.3 | 78.4 |
|  | **Rhinorrhea or sneezing** | 1.3 | 2.2 | 0.7 | 12.8 |
|  | **Rhinorrhea and sneezing** | 0.4 | 0 | 0 | 8.8 |
| **Sensitivity to dust, %** | **No sensitivity** | 78.9 | 69.7 | 52.9 | 32 |
|  | **Rhinorrhea or sneezing** | 23.3 | 27 | 40.7 | 45.6 |
|  | **Rhinorrhea and sneezing** | 0.9 | 3.3 | 6.4 | 22.4 |
| **Sensitivity to tobacco smoke, %** | **No sensitivity** | 97.8 | 98.9 | 86.3 | 92 |
|  | **Rhinorrhea or sneezing** | 1.8 | 1.12 | 11.5 | 7.2 |
|  | **Rhinorrhea and sneezing** | 0.4 | 0 | 2.2 | 0.8 |
| **Sensitivity to cold air, %** | **No sensitivity** | 84.7 | 83.2 | 63.6 | 71.2 |
|  | **Rhinorrhea or sneezing** | 14.9 | 15.7 | 32.1 | 25.6 |
|  | **Rhinorrhea and sneezing** | 0.4 | 1.1 | 4.3 | 3.2 |
| **Sensitivity to weather, %** | **No sensitivity** | 97.8 | 96.6 | 86.4 | 84.8 |
|  | **Rhinorrhea or sneezing** | 1.8 | 1.1 | 10.7 | 12 |
|  | **Rhinorrhea and sneezing** | 0.4 | 2.3 | 2.9 | 3.2 |
| **Use of nasal spray in the last 12 months*, %** |  | 23.3 | 22.5 | 45.7 | 48 |
| **Use of other drug in the last 12 months*, %** |  | 16.2 | 21.4 | 36.4 | 53.6 |

***= p-value<0.001, BMI: Body Mass Index**

Table D. Description of the participants with asthma according to the four classical phenotypes (hypothesis driven)

|  | | |  | **Phenotype 1: No symptoms, no SPT** (n=17) | **Phenotype 2: SPT only** (n=70) | **Phenotype 3: symptoms, no SPT** (n=55) | **Phenotype 4: Symptoms and SPT** (n=259) |
| --- | --- | --- | --- | --- | --- | --- | --- |
| **age, mean± sd** | | | | 47.2±14.2 | 38.2±17.0 | 45.1±17.7 | 35.4±15.0 |
| **Sex, women %** | | | | 58.8 | 41.4 | 52.7 | 46 |
| **Tobacco status, %** | **Non-smoker** | | | 47 | 52.9 | 41.8 | 54.1 |
|  | **Ex-smoker** | | | 41.2 | 25.7 | 30.9 | 18.2 |
|  | **Smoker** | | | 11.8 | 21.4 | 27.3 | 27.8 |
| **BMI, %** | **<20** | | | 0 | 10 | 7.3 | 14.7 |
|  | **[20-25]** | | | 41.2 | 55.7 | 45.5 | 52.1 |
|  | **[25-30]** | | | 41.2 | 21.4 | 30.9 | 23.9 |
|  | **>=30** | | | 17.6 | 12.9 | 16.4 | 9.3 |
| **Nasal symptoms, %** | **Symptoms without eye symptoms** | | |  |  | 40.0 | 21.0 |
|  | **Symptoms with eye symptoms** | | |  |  | 60.0 | 79.0 |
| **Type of nasal symptoms, %** | **ever but not current** | | |  |  | 0 | 0.8 |
|  | **ever and current** | | |  |  | 100 | 99.2 |
| **Report of allergic rhinitis*, %** | | | | 0 | 32.9 | 47.3 | 69.5 |
| **Report of hay fever*, %** | | | | 11.8 | 41.4 | 38.2 | 71.4 |
| **Report of conjonctivitis*, %** | | | | 11.8 | 30 | 32.7 | 55.6 |
| **Report of sinusitis, %** | | | | 41.2 | 47.1 | 60 | 54.1 |
| **Report of eczema*, %** | | | | 41.2 | 42.9 | 25.5 | 56 |
| **Diagnostic of allergy*, %** | | | | 35.3 | 64.3 | 52.7 | 85.7 |
| **BHR=1, %** | | | | 57.1 | 67.7 | 71.0 | 71.8 |
| **FEV1 % predict** | | | | 1.06±0.19 | 0.94±0.22 | 0.97±0.21 | 0.97±0.16 |
| **Sensitivity to hay/flowers*, %** | | **No sensitivity** | | 94.1 | 72.9 | 81.8 | 31.7 |
|  | | **Rhinorrhea or sneezing** | | 5.9 | 14.3 | 5.5 | 29.7 |
|  | | **Rhinorrhea and sneezing** | | 0 | 12.9 | 12.7 | 38.6 |
| **Sensitivity to animals*, %** | | **No sensitivity** | | 100 | 85.7 | 94.5 | 59.9 |
|  | | **Rhinorrhea or sneezing** | | 0 | 8.6 | 3.6 | 18.5 |
|  | | **Rhinorrhea and sneezing** | | 0 | 5.7 | 1.8 | 21.6 |
| **Sensitivity to dust*, %** | | **No sensitivity** | | 82.4 | 60 | 49.1 | 28.6 |
|  | | **Rhinorrhea or sneezing** | | 17.6 | 27.1 | 34.6 | 41.7 |
|  | | **Rhinorrhea and sneezing** | | 0 | 12.9 | 16.4 | 29.7 |
| **Sensitivity to tobacco smoke, %** | | **No sensitivity** | | 100 | 94.3 | 88.9 | 82.6 |
|  | | **Rhinorrhea or sneezing** | | 0 | 5.7 | 7.4 | 12.7 |
|  | | **Rhinorrhea and sneezing** | | 0 | 0 | 3.7 | 4.6 |
| **Sensitivity to cold air, %** | | **No sensitivity** | | 88.2 | 85.7 | 58.2 | 69.1 |
|  | | **Rhinorrhea or sneezing** | | 11.8 | 14.3 | 38.2 | 26.3 |
|  | | **Rhinorrhea and sneezing** | | 0 | 0 | 3.6 | 4.6 |
| **Sensitivity to weather, %** | | **No sensitivity** | | 94.1 | 94.3 | 85.5 | 76.5 |
|  | | **Rhinorrhea or sneezing** | | 5.9 | 5.7 | 9.1 | 13.1 |
|  | | **Rhinorrhea and sneezing** | | 0 | 0 | 5.5 | 10.4 |
| **Use of nasal spray in the last 12 months*, %** | | | | 35.3 | 44.3 | 45.5 | 66.8 |
| **Use of other drug in the last 12 months, %** | | |  | 5.9 | 8.6 | 5.5 | 18.5 |

*= p-value<0.001, BMI= Body Mass Index, BHR: Bronchial HyperResponsiveness (Methacholine test, PD20≤4 mg ), FEV1= Forced Expiratory Volume 1s

**Figure legends**

Fig S1: BIC criterion according to the number of cluster for participants without (Part A) and with (Part B) asthma
